# Neurochronometry of choice-induced preference changes: when do preferences actually change?

**DOI:** 10.3389/fnhum.2023.1222068

**Published:** 2023-06-19

**Authors:** Alina Davydova, Julia Sheronova, Vladimir Kosonogov, Anna Shestakova, Vasily Klucharev

**Affiliations:** Institute for Cognitive Neuroscience, HSE University, Moscow, Russia

**Keywords:** spreading of alternatives, choice-induced preference change, cognitive dissonance, neurochronometry, decision-making, rTMS

## Abstract

According to cognitive dissonance theory, a discrepancy between preferences and actions may lead to the revaluation of preferences, increasing preference for the chosen options and decreasing for the rejected options. This phenomenon is known as the spreading of alternatives (SoA), which results in a choice-induced preference change (CIPC). Previous neuroimaging studies have identified several brain regions that play a role in cognitive dissonance. However, the neurochronometry of the cognitive mechanisms underlying CIPC is a topic of debate. In other words, does it occur during the difficult choice, immediately after the choice, or when people encounter the options again? Furthermore, it remains unclear what is the exact time point, relative to the onset of facing options, either within the choice or after it, when the attitudes start to be revised. We argue that applying online protocols of transcranial magnetic stimulation (TMS), during or immediately after the choice process, could be the most efficient way to better understand the temporal dynamics of the SoA effect. TMS allows for achieving high temporal and spatial resolution, modulating the activity of areas of interest, and examining the causal relationships. Besides, unlike the offline TMS, the online instrument allows tracking of the neurochronometry of attitude change, by varying stimulation onsets and durations with respect to the option stimuli. Based on scrupulous analysis of previous findings, employing online TMS studies of conflict monitoring, cognitive control, and CIPC neuroimaging results, we conclude that the use of online TMS is critical to examine the neurochronometry of CIPC.

## 1. Introduction

Normative decision theories suggest that people’s preferences underlie their choices (Samuelson, 1948; Arrow, 1959), but the cognitive dissonance theory reveals that this is not always the case, pointing out contrary cases (Festinger, 1957). According to Festinger’s theory of cognitive dissonance, choosing between two equally attractive options is considered a difficult decision that may lead to inconsistency between the positive aspects of a rejected option and the negative aspects of a chosen option. This, thereby, induces cognitive dissonance, which is psychologically uncomfortable. Accordingly, such discomfort may motivate people to reduce cognitive dissonance by re-evaluating the negative and positive aspects of the options. A preference for a chosen option tends to increase, whereas a preference for a rejected option tends to decrease. Hereby, cognitive dissonance causes choice-induced preference change (CIPC) and leads to the so-called spreading of alternatives (SoA) (Brehm, 1956; Mann et al., 1969).

The discovery of CIPC has entailed years of behavioral, neuroimaging, and brain stimulation research (for a review, see Enisman et al., 2021). Neuroimaging studies have demonstrated the role of various brain regions in cognitive dissonance, including the dorsolateral prefrontal cortex, the medial prefrontal cortex, the nucleus accumbens (NAcc), the anterior and posterior cingulate cortices, the anterior insula, and the hippocampus (Izuma et al., 2010; Voigt, 2022).

However, the neurochronometry, or the timing of the neurocognitive mechanisms underlying CIPC, still remains under debate. The temporal dynamics of the CIPC and cognitive dissonance-induced preference changes have received relatively little attention, compared to the functional mapping of preference changes. Particularly, it is not clear whether CIPC is implemented and encoded by neural networks, right before making a decision, during the difficult choice, right after the choice, or when people face the selected (or rejected) options again. This issue is not restricted to its specifics, since it might provide insight into the sources of CIPC and disentangle the cognitive dissonance reduction mechanisms from other cognitive processes underlying CIPC, such as value refinement (Voigt, 2022; Lee and Pezzulo, 2023).

## 2. CIPC mechanism timing in reference to the choice process

Neuroimaging studies mostly assume that people revalue or rationalize alternative options only after the choice was made and filed in memory, or after addressing the options again (Izuma et al., 2010, 2015; Kitayama et al., 2013; Mengarelli et al., 2015; Chammat et al., 2017). In other words, according to this view, the central nervous system continuously detects that preferences are not aligned with previous choices and modifies preferences accordingly, leading to the SoA. Importantly, the basic behavioral SoA paradigm consists of at least three stages: *Rating 1*, the first stage, where participants rate items for the first time; *Choice*, the second stage, where they choose between similarly rated pairs of items; and *Rating 2*, the third stage, where participants rate all the items again. Interestingly, in most neuroimaging studies, the Choice stage has been largely ignored. Instead, previous neuroimaging studies have predominantly focused on the Rating 2 stage as the critical phase for preference change (Izuma et al., 2010, 2015; Mengarelli et al., 2015; Chammat et al., 2017). For example, pioneering studies by Izuma et al. (2010) and Qin et al. (2011) analyzed functional magnetic resonance imaging (fMRI) data, only during both Rating tasks, and not during the Choice task.

Yet, a limited number of fMRI and electroencephalography (EEG) studies have specifically focused on neural activity during difficult choices (Nakao et al., 2016; Colosio et al., 2017; Voigt et al., 2019) and have shown that preference changes might already be implemented during the Choice stage of the SoA paradigm. For example, Voigt et al. (2019) demonstrated that preference changes were predicted by the activity in the left dorsolateral prefrontal cortex and precuneus while making difficult choices. The authors later theorized their results into a model, assuming that the need to choose elicits an adaptation mechanism that adjusts preferences and further reconstructs the value-based choice (Voigt, 2022). Thus, identifying the neural signature of the moment of the CIPC launch would help to clarify further discussion of the exact preference change neural mechanisms. In this vein, an EEG study demonstrated that difficult choices during the Choice stage triggered error-related negativity, which is correlated with the reevaluation of the alternatives (Colosio et al., 2017). Recent fMRI studies have shown that the activity of the medial cortices and NAcc during difficult choices predicts subsequent preference changes (Jarcho et al., 2011; Kitayama et al., 2013). Moreover, eye-tracking findings suggested that the fixations pattern during the Choice stage served as good predictors of the direction and amplitude of preference changes (Voigt et al., 2019). Importantly, recent computational studies of CIPC link attitude change, with a learning rate that is updated exactly during choices (Vinckier et al., 2019; Zhu et al., 2021). The reinforcement learning and Bayesian models showed that such a learning rate update was the best predictor of participants’ behavior. Moreover, some quantitative studies have demonstrated not only the CIPC timing but also the computational mechanisms related to temporal dynamics (Lee and Daunizeau, 2020, 2021).

Despite significant progress in CIPC research, there is no consensus on the neurochronometry underlying CIPC. It is possible that the post-decisional SoA may occur right before, during the choice, right after the choice, or (and) later, when people face the options again. Here, we suggest that an application of the online protocols of transcranial magnetic stimulation (TMS), not only during Rating 2, but also during the Choice task, may lead to a deeper understanding of the temporal dynamics underlying the SoA effect.

## 3. Online TMS instrument for CIPC exploration

Unlike other neuroimaging methods, online TMS gives an opportunity to activate and deactivate regions of interest by stimulating them precisely in time and space. TMS is often used in causal brain mapping, i.e., in finding out a causal relationship between certain brain areas and the brain function. Such stimulation can be offline or online. Online TMS has certain advantages compared with offline TMS. Importantly, to clarify the temporal neural dynamics underlying the SoA, one could vary the onsets of TMS to determine the time windows, when TMS can efficiently eliminate the SoA. However, the offline brain stimulation that has been used in previous studies, does not allow us to trace the neurochronometry of cognitive dissonance in the necessary detail. Online TMS can be effectively used to infer the timing and location of (cortical) neuronal events underlying changes in attitudes during different stages of the SoA paradigm. Interestingly, TMS studies are less sensitive to the behavioral artifacts associated with SoA paradigms, which may reveal already existing preferences, instead of a result of a shift in preferences (Chen and Risen, 2010). Since the above-mentioned artifacts are similar in the experimental and control TMS conditions, all behavioral differences across conditions can be attributed only to the effect of the TMS on the neural events underlying the SoA. However, certain limitations, such as electric field distribution modeling, complex neuronal response, and the TMS confounding effects, drive the need for subtle and accurate elaboration and revision of the TMS protocols (Hobot et al., 2021; Siebner et al., 2022).

## 4. Effectiveness of different online TMS onsets in cognitive conflict tasks

Surprisingly, online TMS protocols have never been applied to dissonance-inducing tasks, which provides no reference points in terms of the timing of an effective online TMS protocol. Nevertheless, based on similar behavioral paradigms (Friehs et al., 2020; Parris et al., 2021), it is possible to speculate on the onsets of online TMS which would be effective in eliminating the SoA. We can base future online TMS studies of the cognitive dissonance on the previous results of the typical conflict-inducing paradigms, namely, the Stroop task, flanker paradigm, and Simon tasks (for a review see Olk et al., 2015), since they also involve conflict detection and resolution mechanisms. It should be emphasized that various cortical areas have been associated with conflict detection and cognitive control. Hence, here, we ignore online TMS studies that target brain zones, mostly related to visuomotor integration, but instead focus on TMS studies of the prefrontal cortex.

Some studies have used TMS to intervene in the process of conflict monitoring by downregulating the prefrontal cortex, immediately after the stimulus onset, without any delay (Soutschek et al., 2013; Zhao et al., 2018; Friehs et al., 2020), or even 100 ms before the stimulus onset (Taylor et al., 2007). Other studies have hindered cognitive conflict processing by stimulating the cortex at 100 ms (Obeso et al., 2013), or at 200 ms (Hayward et al., 2004, 2007), after the stimulus onset. However, these TMS studies did not allow for a comparison of the effect of different delays and included stimulation at only one latency. TMS studies of the Stroop effect increased the error frequency, using various time ranges of pulse delivery: from 125 to 175 ms (Cai et al., 2012), from 0 to 100 ms (Chen et al., 2009), and from 20 to 200 ms (Masina et al., 2018) after the stimulus onset. Nevertheless, there was no pulse timing effect in these studies which can be explained by affecting different levels of conflict monitoring, since other brain regions, including the primary motor cortex, become active due to the prefrontal area modulation (Obeso et al., 2013). Combined EEG-TMS studies are of particular interest because they enable additional control of the effect of TMS pulses’ delay by monitoring task-induced neurophysiological activity. Verleger et al. (2009) delivered TMS pulses at four latencies with a 30 ms interval, starting from the 281 ms peak of the flanker-evoked brain activity (lateralized readiness potential (LRP) waveform peak). They found that the 311 ms latency (30 ms after the peak, 281 ms after the flanker onset, and 174 ms after the target one) of the TMS pulses is critical for cognitive conflict resolution. Using this protocol, researchers discovered that in trials where flankers are incompatible with targets, the LRP amplitude is positively correlated with the amplitude of the motor-evoked potential, which can give inference into the start of switching to the correct response in conflict trials.

## 5. Optimal timing of CIPC TMS studies

To the best of our knowledge, there are no online TMS studies of CIPC, and for now, one can only speculate about the optimal timing of online TMS stimulation, based on relevant EEG studies. Interestingly, Colosio et al. (2017) demonstrated that the neural activity associated with difficult choices was distributed frontocentrally and peaked at ∼60 ms after the button press. The CIPC neural response was similar to error-related negativity, which peaks in the range of 60–120 ms after an error has been made (Gehring et al., 1993). Meanwhile, Nakao et al. (2016) linked the preference change to frontocentral beta and gamma power, at the time interval of around 400 ms after the decision. The discovered early responses give a clue to the CIPC timing in reference to the choice stage, and to the promising opportunity to analyze the neural signature of CIPC during the choice.

In addition, we would like to suggest paying attention not only to studies with the choice between equally attractive options, but also to research other non-reinforced preference changes, such as the ones induced by social influence. A magnetoencephalography (MEG) study of recommendation-based social influence on preference change (Irani et al., 2022), found the evoked activation in the 68–245 and 320–998 ms time windows after the conflict trials’ onset, in which the individual’s opinion was inconsistent with the opinion of the group. Irani et al. (2022) consider the early time window to be a reflection of the negative emotion of pressure to change the initial preference in the face of a social rejection threat which is compatible with the cognitive dissonance theory (Festinger, 1957) in creating uncomfortable feelings and a strong motivation to retrieve an acceptable state.

## 6. Conclusion

Overall, previous studies of conflict monitoring have demonstrated a large variance in the timing of online TMS protocols. Besides, the choice of the timing for online TMS was rarely supported by the neuroimaging data with high temporal resolution, such as EEG and MEG. Although it is well known that the effects of online TMS on cognitive processing are latency dependent, 90% of online TMS studies applied stimulation simultaneously with stimuli onsets (for a meta-analysis see Beynel et al., 2019). Here, we would like to stress that a proper understanding of the neural mechanisms of CIPC calls for a set of online TMS interventions, applied to different brain sites and time windows, in order to clarify their functional role in CIPC. Initial TMS studies may synchronize stimulation with the choices (Choice stage), or with the onset of the second presentation of the rejected or selected options (Rating 2 stage). Follow-up studies may vary the delay between the critical stages of the choice-induced paradigm and onsets of TMS pulses, in order to study the neurochronometry of the CIPC more precisely. It is also critical that the CIPC researchers associate their hypotheses regarding CIPC temporal dynamics with an attempt to understand which cognitive processes manifest behaviorally as the preference change, and to disentangle between them.

In our opinion, the online TMS that is able to dysregulate neurocognitive mechanisms at different time points can be an optimal tool for resolving the current discussion about the actual onset of CIPC. Using different protocols of online TMS, we can clarify whether the revaluation of alternatives occurs during choices, or later, when the person faces the rejected or selected options again. Finally, by combining TMS with EEG and fMRI, we will be able to further characterize brain connectivity and temporal dynamics underlying CIPC.

## Author contributions

AD and JS: conceptualization, investigation, writing—original draft, and review and editing. VKo, AS, and VKl: conceptualization and writing—review and editing. All authors contributed to the article and approved the submitted version.
